# Genome-Wide Identification and Expression Divergence of *CBF* Family in *Actinidia arguta* and Functional Analysis of *AaCBF4* Under Cold Stress

**DOI:** 10.3390/life15020227

**Published:** 2025-02-04

**Authors:** Sumei Li, Qina Zhang, Zhenzhen Zhang, Peng Zhang, Congcong Li, Leiming Sun, Jinbao Fang, Ran Wang, Feng Wei, Yukuo Li, Miaomiao Lin, Xiujuan Qi

**Affiliations:** 1 School of Agricultural Sciences, Zhengzhou University, Zhengzhou 450001, China; 15615642056@163.com (S.L.); zhang_qina99@163.com (Q.Z.);; 2 National Key Laboratory for Germplasm Innovation & Utilization of Horticultural Crops, Zhengzhou Fruit Research Institute, Chinese Academy of Agricultural Sciences, Zhengzhou 450009, China; 3 National Key Laboratory of Cotton Bio-Breeding and Integrated Utilization, Institute of Cotton Research, Chinese Academy of Agricultural Sciences, Anyang 455000, China; 4 Zhongyuan Research Center, Chinese Academy of Agricultural Sciences, Xinxiang 453500, China

**Keywords:** kiwifruit, *CBF* gene family, low-temperature stress

## Abstract

The C-repeat binding factors (CBFs) gene is essential for plants’ cold response, which could not only be induced by the inducer of CBF expression (ICE) genes but also activated the expression of the cold-regulated (COR) gene, thereby participating in the ICE-CBF-COR cold response pathway. However, this gene family and its functions in *Actinidia arguta* remain unclear. In this study, whole-genome identification and functional analysis of *CBF* family members in *A. arguta* were performed. Eighteen *CBF* genes, which were located on four chromosomes and had five tandem repeats, were identified. The proteins encoded by the genes were predicted to be located in the nucleus and cytoplasm. The results of the promoter cis-acting element analysis revealed light response elements, low-temperature response elements, and hormone (methyl jasmonate, gibberellin, salicylic acid, etc.) response elements. We analyzed collinearity with other kiwifruit genomes, and, interestingly, the number of *CBF* family members differed across geographic locations of *A. arguta*. RT-qPCR revealed that the expression of the *CBF* gene family differed under low-temperature treatment; specifically, we observed differences in the expression of all the genes. Based on phylogenetic relationships and RT-qPCR analysis, the expression of *AaCBF4.1* (*AaCBF4*) was found to be highly upregulated, and the function of this gene in cold resistance was further verified via overexpression in transgenic Arabidopsis. *AaCBF4*-overexpressing plants showed higher tolerance to cold stress, showing a higher germination rate, higher chlorophyll content and lower relative electrolyte leakage. In addition, compared with the wild-type Arabidopsis, the overexpressing plants exhibited significantly reduced oxidative damage due to the reduction in reactive oxygen species production under cold stress. Therefore, *AaCBF4* plays an important role in improving the cold resistance of *Actinidia arguta* and can be further used to develop kiwifruit germplasm resources with strong cold resistance.

## 1. Introduction

Temperature is an important factor affecting the growth and geographical distribution of plants [1,2,3]. With the trend toward an increase in global warming, extreme weather is occurring more frequently, which has multiple implications and poses risks for the natural environment and social ecology [4,5]. For example, low temperatures, especially those experienced during early spring chilling, late autumn chilling, and winter chilling, could have a considerable effect on the yield and quality of kiwifruit [6]. Low-temperature stress, which can be divided into cold stress (0–15 °C) and freezing stress (<0 °C) [7], poses severe threats to the growth, development, and metabolism of plants. When plants are exposed to low temperatures for a long period, they undergo some physiological and biological changes that increase their resistance to cold stress, which is referred to as cold acclimation [8,9,10]. Cold stress can cause metabolic disorders in plant cells, damage membrane systems, and reduce photosynthesis, thereby affecting plant growth and development. At low temperatures, the permeability of plant cell membranes increases, leading to electrolyte leakage, and lipid peroxidation of the membranes generates a large amount of reactive oxygen species and malondialdehyde, resulting in damage. To resist the adverse effects of cold stress, plants synthesize osmotic adjustment substances (soluble sugars and soluble proteins) and antioxidant enzymes (superoxide dismutase, catalase, and peroxidase) to protect the biomembrane system, thereby increasing the tolerance of plants to low temperatures [11,12].

Recently, the molecular mechanisms by which plants acquire cold resistance through cold acclimation have been extensively studied. Inducer of CBF expression1 (ICE1) was a Basic Helix-Loop-Helix (bHLH) transcriptional activator that induced C-repeat binding factors (*CBFs*) genes expression and exerted the function during cold acclimation by binding to the promoter of the *ICE* gene. And the rapid upregulation of the cold-regulated (*COR*) gene was a key part of the response to low-temperature stress in plants [7,9,13]. To date, the signaling pathway most clearly identified as being involved in the response to cold stress is the ICE-CBF-COR pathway [14,15]. C-repeat/DREB factor (CBF) proteins have been identified as critical transcription factors that can respond to low temperatures [16,17]. CBF, also known as the dehydration-responsive-element-binding (DREB) protein, contains a conserved AP2 (APETALA2) domain, which belongs to the AP2/ERF family [18,19]. To date, *CBF* genes have been identified in several species, including *Arabidopsis thaliana* [9], *Lolium perenne* [20], *Malus domestica* [21], *Camellia sinensis* [22], and *Vitis vinifera* [23], and their functions in regulating plant cold tolerance have been identified.

In *A. thaliana*, *AtCBF1*, *AtCBF2*, and *AtCBF3* were identified as the active regulatory factors that respond to cold stress [24]. In apples (*M. domestica*), cold stress induces the interaction of *NAC104* with the promoter of *CBF1/3* to activate *COR* genes to regulate cold tolerance [7]. In grape (*Vitis amurensis*), *VaMYC2* interacts with and activates *VaCBF1* to increase resistance to low temperatures [25].

Kiwifruit, which belongs to *Actinidia* Lindl, in the Actinidiaceae family, is rich in a variety of nutrients; has a high nutritional, medicinal, and economic value; and is distributed mainly in tropical and temperate regions [26,27]. However, abiotic stress, particularly cold stress, has devastating effects on the growth and development of kiwifruit, which may lead to decreased plant yield and, in the worst case, to plant death. Our previous study revealed that *AaCBF4* can bind to the *AaBAM3.1* promoter and regulate the expression of *AaBAM3.1* to increase cold resistance when *Actinidia. arguta* is subjected to low-temperature stress [28]. However, there has been no systematic study of the *CBF* gene family in *A. arguta*. The publication of the genome data of autotetraploid *A. arguta* has provided the possibility to analyze the *CBF* gene family at the genomic level [29].

In *A. arguta*, the *CBF* gene family is a complex gene family containing multiple members that differ in their expression patterns under low-temperature stress, and this difference may reflect their different functions and mechanisms of action in cold resistance in plants. The acquisition of genomic data of *A. arguta* provides a basis for the analysis of its gene family, which helps to further understand the gene functions and regulatory mechanisms. In this study, we identified and analyzed the *CBF* gene family in the genome of *A. arguta*. The expression of the *CBF* gene family members of *AaCBF4.1* could be induced by low temperatures, indicating that it may be involved in the response and adaptation of plants to low-temperature stress. Moreover, using gene sequence alignment, we found that this gene was identical to the *AaCBF4* gene of the *AaCBF4*-*AaBAM3.1* module pathway studied in the previous stage. Therefore, we chose *AaCBF4* as a candidate gene associated with cold resistance. In addition, by overexpressing *AaCBF4* in *A. thaliana*, we found that *AaCBF4* had a positive regulatory effect on the response to low temperatures. The results of this study provide a foundation for further research on the regulatory mechanisms of *CBF* gene family members, offering new strategies for the genetic improvement and breeding of cold-resistant kiwifruit.

## 2. Materials and Methods

### 2.1. Plant Materials

The *A. arguta* plants were obtained from Zhengzhou Fruit Research Institute (113°42′ E, 34°42′ N), Zhengzhou, Henan Province, China. The dormant branches of the kiwifruit were collected and treated at low temperatures for 0 h, 2 h, 4 h, 6 h, and 8 h. In addition, the flowers, branches, dormant branches, leaves, and roots of the kiwifruit were collected. The samples were stored at −80 °C for subsequent qRT‒PCR analysis of the low-temperature expression of the *CBF* gene family and the tissue-specific expression of *AaCBF4*.

### 2.2. Identification of the CBF Genes

The genomic data of *A. arguta* were downloaded from GSA “https://bigd.big.ac.cn/gsa/ (accessed on 8 October 2024)”with BioProject ID PRJCA010661 under the accession GWHBJWW00000000. Six CBF protein sequences (gene IDs: AT4G25490.1, AT4G25470.1, AT4G25480.1, AT5G51990.1, AT1G12610.1, and AT1G63030.1) of *A. thaliana*, which were downloaded from TAIR “https://www.arabidopsis.org/ (accessed on 8 October 2024)”, were used as a reference. Using *A. thaliana* as a reference, BLAST analysis revealed the presence of *CBF* genes in *A. arguta* (e-value < 1 × 10^−10^). The file for the AP2 structural domain (PF00847) was downloaded from InterPro “https://www.ebi.ac.uk/interpro/ (accessed on 9 October 2024)” to identify protein sequences with the AP2 domain (e-value < 1 × 10^−10^) by HMMER analysis in TBtools 2.152. The BLAST-extracted sequence was the same as the HMMER protein sequence, and the IDs of the candidate *CBFs* were eventually derived after deleting the *CBFs* without the conserved amino acid sequences (PKK/RP-AGRxKFxETRHP and DSAWR). After a manual analysis of the predicted *CBF* genes in the NCBI database “https://www.ncbi.nlm.nih.gov/Structure/bwrpsb/bwrpsb.cgi (accessed on 9 October 2024)” using Batch CD-Search to search for the AP2 domain, 18 potential *CBFs* were identified. ExPASy “https://www.expasy.org/ (accessed on 15 October 2024)”was used to determine biochemical parameters, such as the molecular weight (MW) and isoelectric point (pI). Then, Cell-PLoc 2.0 tool “http://www.csbio.sjtu.edu.cn/bioinf/Cell-PLoc-2/ (accessed on 15 October 2024)” was subsequently used to predict the subcellular localization of *AaCBF* genes products.

### 2.3. Phylogenetic Tree Analysis

Databases such as NCBI “https://www.ncbi.nlm.nih.gov/ (accessed on 8 October 2024)” and TAIR “http://www.arabidopsis.org/ (accessed on 8 October 2024)” were used to retrieve proteins sequences of CBF from diverse species (*Arabidopsis thaliana*: AtCBF1 (AT4G25490.1), AtCBF2 (AT4G25470.1), AtCBF3 (AT4G25480.1), AtCBF4 (AT5G51990.1), AtDDF1 (AT1G12610.1), and AtDDF2 (AT1G63030.1); *Vitis vinifera*: VvCBF1 (AIL00537.1), VvCBF2 (AIL00651.1), VvCBF3 (AIL00738.1), and VvCBF4 (AIL00828.1); and *Camellia sinensis*: CsCBF1 (EU563238.1), CsCBF2 (KC702795.1), CsCBF3 (MH017428.1), CsCBF4 (KF988866.1), CsCBF5 (MH165878.1), and CsCBF6 (MN544638.1)). The evolutionary relationships of the above CBF genes with CBF genes members of *A. arguta* were analyzed. With the MEGA 11 software, a phylogenetic tree was created by applying a maximum likelihood method. The phylogenetic tree was subsequently visualized via Evolview “https://www.evolgenius.info/evolview/ (accessed on 10 October 2024)”.

### 2.4. Gene Structure, Conserved Domain, and Conserved Motif Analysis

Through the genome file and genome annotation file, the position and length of the *AaCBF* genes on the chromosome were determined, and the positions of introns and exons were predicted. Finally, TBtools was used to identify and visualize the structures of the *CBF* genes in *A. arguta*. The conserved motif of *A. arguta* was investigated via MEME “https://meme-suite.org/meme/ (accessed on 18 October 2024)”. The files of the conserved domains of the *AaCBFs* were downloaded from the Conserved Domain Database (CDD) of the NCBI website. Subsequently, the phylogenetic tree, gene structure, conserved domains, and conserved motifs of the *CBF* gene family were all visualized with TBtools 2.152.

### 2.5. Cis-Acting Element Analysis

The promoter sequences, which were found in the 2000 bp stretches upstream of the *CBF* gene CDSs, were extracted via TBtools. Afterward, TBtools was utilized to visualize the cis-acting elements that were found via Plant CARE “https://bioinformatics.psb.ugent.be/webtools/plantcare/html/ (accessed on 19 October 2024)”.

### 2.6. Chromosomal Mapping and Collinearity

The chromosomal locations of *CBFs* in *A. arguta* were analyzed on the basis of the genome sequence and genome annotation data, and through TBtools visualization, it was found that the *CBFs* were mapped to 14 chromosomes based on their actual positions. TBtools was subsequently used to demonstrate collinearity analysis among three species. The genomes of *Actinidia. chinensis* and *Actinidia. eriantha* were downloaded from the kiwifruit genome database “http://kiwifruitgenome.org/ (accessed on 21 October 2024)”, while the genomes of *A. arguta* were downloaded from GSA “https://bigd.big.ac.cn/gsa/ (accessed on 8 October 2024)” with BioProject ID PRJCA010661 under the accession GWHBJWW00000000 and from the NGDC database “https://ngdc.cncb.ac.cn/ (accessed on 21 October 2024)” with the accession number PRJCA022944.

### 2.7. Total RNA Isolation and RT‒qPCR Analysis

The plant tissue samples stored at −80 °C were ground into fine powder (0.2 g) in liquid nitrogen, and total RNA was extracted according to the instructions of the Quick RNA Isolation Kit (Waryong, Beijing, China). The cDNA was synthesized via reverse transcription using a First Strand cDNA Synthesis Kit (TOYOBO, Shanghai, China), and the obtained cDNA was diluted five times in nuclease-free water. In this study, the primers were devised via Primer Premier 5 and were designed via agarose gel electrophoresis (Table S1). RT‒qPCR was performed On A Roche 480 Real-time PCR machine using the SYBR qPCR SuperMix Plus (Novoprotein, Shanghai, China). *AaActin* (Gene ID: Aa2Ag122168.1) from *Actinidia* was selected as a reference gene. The RT‒qPCR was completed with three technical repeats, and the 2^–ΔΔCt^ method was adopted to calculate the relative expression levels of the examined genes.

### 2.8. Construction of Transgenic Arabidopsis Lines

The open reading frames (ORFs) of AaCBF4 were subsequently amplified from the cDNA of *A. arguta* (KL) leaves with the specific primers AaCBF4-ORF-F/R (Table S1). To explore the function of the *AaCBF4* gene in *A. thaliana*, the pBI121-AaCBF4 overexpression vectors was constructed by inserting the CDSs of *AaCBF4* into the expression vector pBI121 between the restriction sites of SalI and BamHI of the CaMV35S promoter via the ClonExpress II One Step Cloning Kit (Vazyme, Nanjing, China). *A. thaliana* was cultured in soil supplemented with a 2:2:1 ratio of nutrient soil, vermiculite, and perlite and then incubated in a growth chamber at 24 °C and 65% relative humidity under a 16 h/8 h (day/night) photoperiod. The vectors were introduced into WT plants (Col-0) via an inflorescence infection method. The transgenic plants were subsequently screened for positive plants on a 1/2 MS medium supplemented with 100 mg/L kanamycin until the progeny (T3 generation) showed no trait segregation and were confirmed to be pure. pBI21-GFP-AaCBF4-F/R primers were used to amplify specific bands using PCR with the DNA of the leaves of positive transgenic plants used as a template to indicate that the pBI121-AaCBF4 overexpression vector was successfully inserted into the transgenic plants. With the *A. thaliana AtActin* gene (NCBI NM_103814.4) used as an internal reference gene, the expression level of *AaCBF4* in the leaves of the above plants was identified by RT‒qPCR with AaCBF4-F/R primers. Three T3-transformed plants with a high expression of *AaCBF4* were selected for further verification of the gene function (Figure S2).

### 2.9. Assessment of Chilling Tolerance

To assess the cold tolerance of the WT plants and the AaCBF4 overexpression plants, 4-week-old Arabidopsis plants were treated at −2 °C for 4 h to observe their phenotypes and determine their physiological indices. The leaves of each plant lines were weighed to 0.2 g and placed in 10 mL distilled water, and the conductivity was determined as E1 after 2 h of oscillation at 200 rpm at room temperature via a conductivity meter (Lei Zhi, Shanghai, China); then, the sample was boiled for 30 min, and the conductivity was measured as E2 after the sample was cooled to room temperature. The relative electrolyte leakage (REL) was calculated via the following formula: REL (%) = E1/E2 × 100. The plants were incubated in darkness for 20 min, after which the Fv/Fm of the plant was determined via Imaging WinGegE 2.41 software on an IMAGING-PAM chlorophyll fluorometer (Walsz, Evertrich, Germany). The levels of malondialdehyde (MDA), proline (Pro), O_2_^−^, H_2_O_2_, and antioxidant enzymes, such as the catalase (CAT) and superoxide dismutase (SOD), were determined via appropriate detection kits (Solarbio, Beijing, China) following the instructions. To evaluate the accumulation of H_2_O_2_ and O_2_^−^ under cold treatment, whole plants were washed with distilled water and immersed in the diaminobenzidine (DAB) or nitro blue tetrazolium (NBT) staining solution. The test tubes were subsequently wrapped in aluminum foil and incubated overnight at room temperature; then, the leaves were soaked in ethanol and heated to remove chlorophyll.

### 2.10. Statistical Analysis

All the experiments were repeated three times independently. The SPSS software (SPSS Statistics 23.0) was used for statistical analysis of the data. Statistically significant differences were identified via Duncan’s multiple-comparison test for sample comparisons, and differences were considered statistically significant at *p* < 0.05 (*), *p* < 0.01 (**), and *p* < 0.001 (***).

## 3. Results

### 3.1. Identification of the AaCBF Family

**Figure 1:**
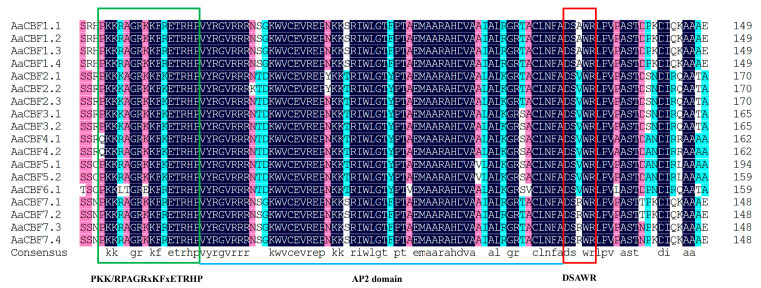
Multiple-sequence alignment of the amino acid sequences of AaCBFs. The two characteristic motifs, the PKK/RPAGRxKFxETRHP and DSAWR motifs, are shown in the box, and the conserved AP2 domain is represented by in the horizontal line.

Eighteen protein sequences in total were verified to belong to the *AaCBF* gene family via an ortholog BLAST search against *A. arguta,* utilizing known CBF amino acid sequences of *A. thaliana* and an HMM analysis of conserved AP2 domain (Figure 1). These CBFs presented different lengths of coding sequences (CDSs). AaCBF5.1 presented the longest CDS of all the *CBFs*, measuring 826 bp, with the longest encoded *CBF* protein sequence, consisting of 270 amino acids. The protein sequence of AaCBF3.1 was the shortest, with 202 amino acids, with a CDS length of 619 bp. An analysis of the physicochemical properties of the *CBF* proteins revealed that the isoelectric point (pI) of the proteins varied from 4.95 to 6.00, whereas their molecular weight (MW) ranged from 22,291.74 kDa to 30,354.12 kDa (Table 1). According to the subcellular localization prediction results, ten *CBF* proteins were found in both the cytoplasm and the nucleus, whereas eight *CBF* proteins were localized in only the nucleus. These findings revealed that the *AaCBF* proteins could function under a variety of conditions.

### 3.2. Phylogenetic Relationships of the CBF Gene Family

**Figure 2:**
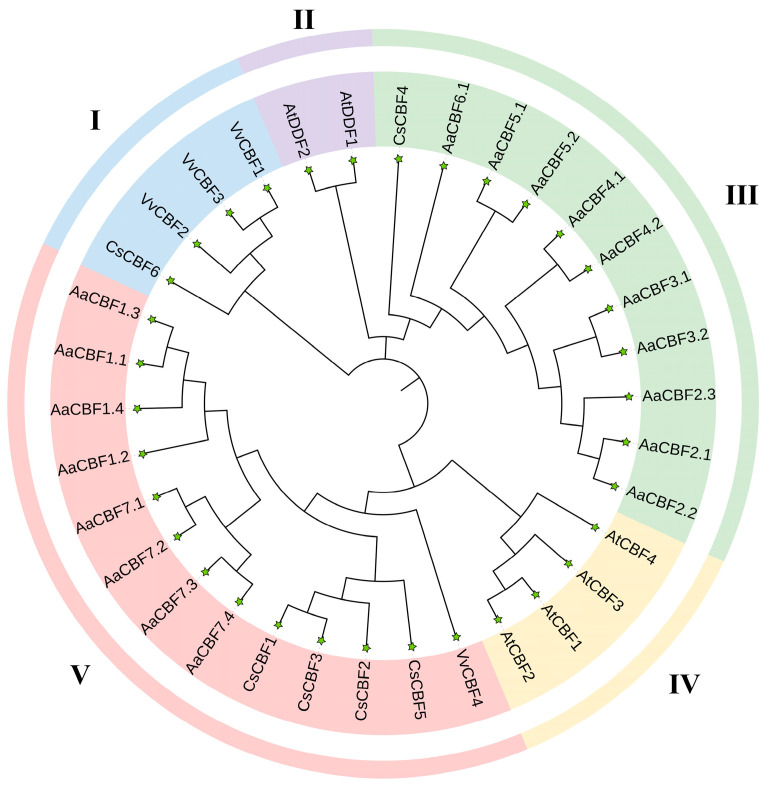
Phylogenetic tree of the *CBF* proteins from different genera. Different colors represent the different homologous clusters. At: *Arabidopsis thaliana* (AtCBF1, AT4G25490.1; AtCBF2, AT4G25470.1; AtCBF3, AT4G25480.1; AtCBF4, AT5G51990.1; AtDDF1, AT1G12610.1; and AtDDF2, AT1G63030.1); Aa: *Actinidia arguta* (Table 1); Cs: *Camellia sinensis* (CsCBF1, EU563238.1; CsCBF2, KC702795.1; CsCBF3, MH017428.1; CsCBF4, KF988866.1; CsCBF5, MH165878.1; and CsCBF6, MN544638.1); and Vv: *Vitis vinifera* (VvCBF1, AIL00537.1; VvCBF2, AIL00651.1; VvCBF3, AIL00738.1; and VvCBF4, AIL00828.1).

The phylogenetic tree was built using the sequences of a total of twenty-four CBF proteins, comprising the six proteins from *A. thaliana* (*AtCBFs*), four proteins from *V. vinifera* (*VvCBFs*), six proteins from *C. sinensis* (*CsCBFs*), and eighteen proteins from *A. arguta* (*AaCBFs*). These *CBFs* were clustered into five groups, namely, Group I, Group II, Group III, Group IV, and Group V, of which Group V was the largest group, followed by Group III. The *CBFs* of *A. arguta* were clustered in Group III and Group V. In addition, Group V contained eight *AaCBFs*, and Group III contained ten *AaCBFs* (Figure 2). The *AaCBFs* from Group III were in the same branch as *CsCBF4*, and the *AaCBFs* from Group V were in the same branch as *CsCBF1*, *CsCBF2*, *CsCBF3*, *CsCBF5*, and *VvCBF4*, which have been reported as important factors in the cold stress response of plants. These results suggested that these *CBF* genes may have similar functions and could play roles in the resistance of the plants to cold stress.

### 3.3. Gene Structures, Conserved Domains, and Conserved Motifs

**Figure 3:**
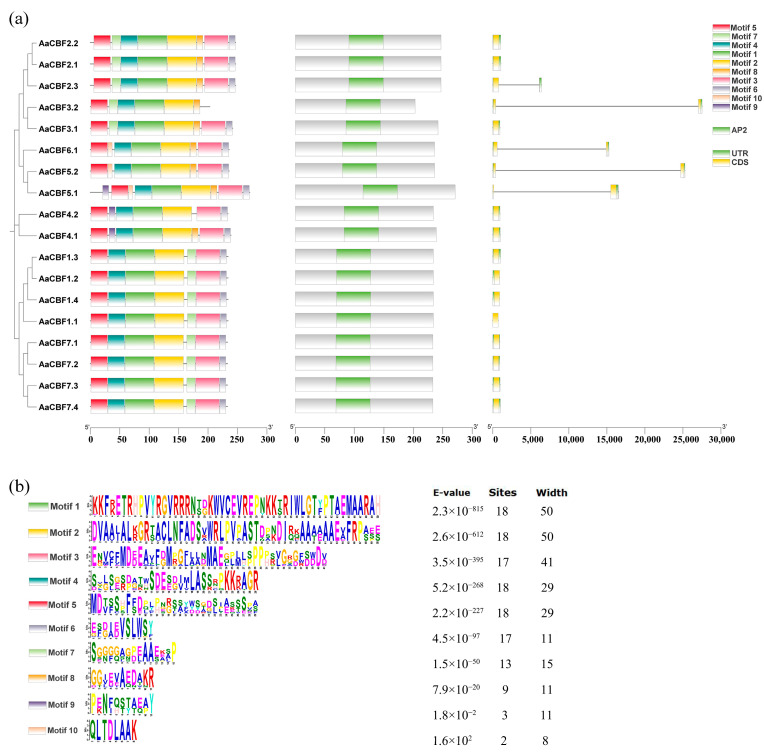
Analysis of the conserved motifs, conserved domains, and gene structures of the *AaCBFs* family in *A. arguta*. (**a**) Conserved motifs, conserved gene domains, and gene structures of *AaCBFs* and (**b**) amino acid sequences of motifs.

The structures, domains, and motifs of the *CBF* genes in *A. arguta* were analyzed, and these eighteen *CBFs* were ordered according to the phylogenetic tree. Six to nine conserved motifs were found in the *AaCBF* genes. Motifs 5, 4, 1, and 2 were discovered in all the *AaCBFs*, suggesting that the members of the *CBF* family in *A. arguta* were relatively well conserved. Motif 9 was found in AaCBF5.1, AaCBF4.1, and AaCBF4.2, whereas motif 10 was found in AaCBF5.1, AaCBF5.2, and AaCBF6.1. Furthermore, AaCBF3.1 did not contain motif 6. Motif 7 was absent in AaCBF5.1, AaCBF5.2, and AaCBF4.2. Motif 8 was not found in Group V (Figure 3a,b), which indicated that different motifs may be associated with the different functions of the *CBF* genes.

Analysis of the *CBF* gene domains revealed that the *AaCBF* genes contained a highly conserved AP2 structural domain (Figure 3a), which suggested that the *CBF* genes of *A. arguta* were conserved during the evolutionary process. In addition, according to the analysis of gene structure, most *AaCBFs* contained only one exon and one intron. However, the sequences of five *CBFs*, *AaCBF2.3*, *AaCBF5.1*, *AaCBF3.1*, *AaCBF5.2*, and *AaCBF6.1*, covered two exons and one intron (Figure 3b). These results indicated that the gene structures were similar among the *CBF* genes that were grouped on the same branch.

### 3.4. Cis-Elements in the Promoters of AaCBF Genes

**Figure 4:**
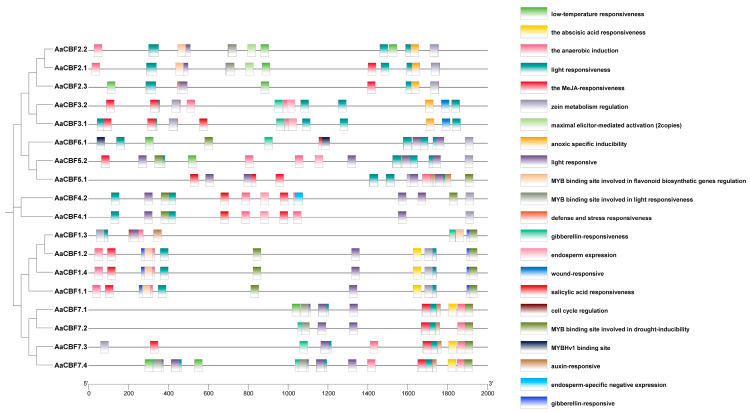
Cis-acting elements in *CBF* promoters. Different colors represent different cis-elements.

The promoter is an important component of the gene expression, and its main function is to regulate the initiation and strength of gene expression [30]. Therefore, to investigate the biological roles of the promoters of the *AaCBF* genes, we examined the cis-elements in the promoter regions (the 2 kb upstream sequences) of each of the *AaCBF* genes via the PlantCARE Database. The promoters of 18 *AaCBF* genes contained a total of 31 distinct cis-elements. Low-temperature response elements were detected in the promoters of *AaCBF2.1*, *AaCBF2.2*, *AaCBF2.3*, *AaCBF5.2*, *AaCBF6.1*, *AaCBF7.1*, and *AaCBF7.4*. The promoters of all *CBF* genes had a large number of light-responsive elements. The promoters of the *CBF* genes also contained elements associated with hormone responsiveness, such as abscisic-acid-responsive elements, MeJA-responsive elements, gibberellin-responsive elements, salicylic-acid-responsive elements, and auxin-responsive elements (Figure 4). The identification of these cis-acting elements revealed that the *CBF* genes were crucial in the treatment of abiotic stress in *A. arguta*.

### 3.5. Chromosomal Location and Synteny Analysis

**Figure 5:**
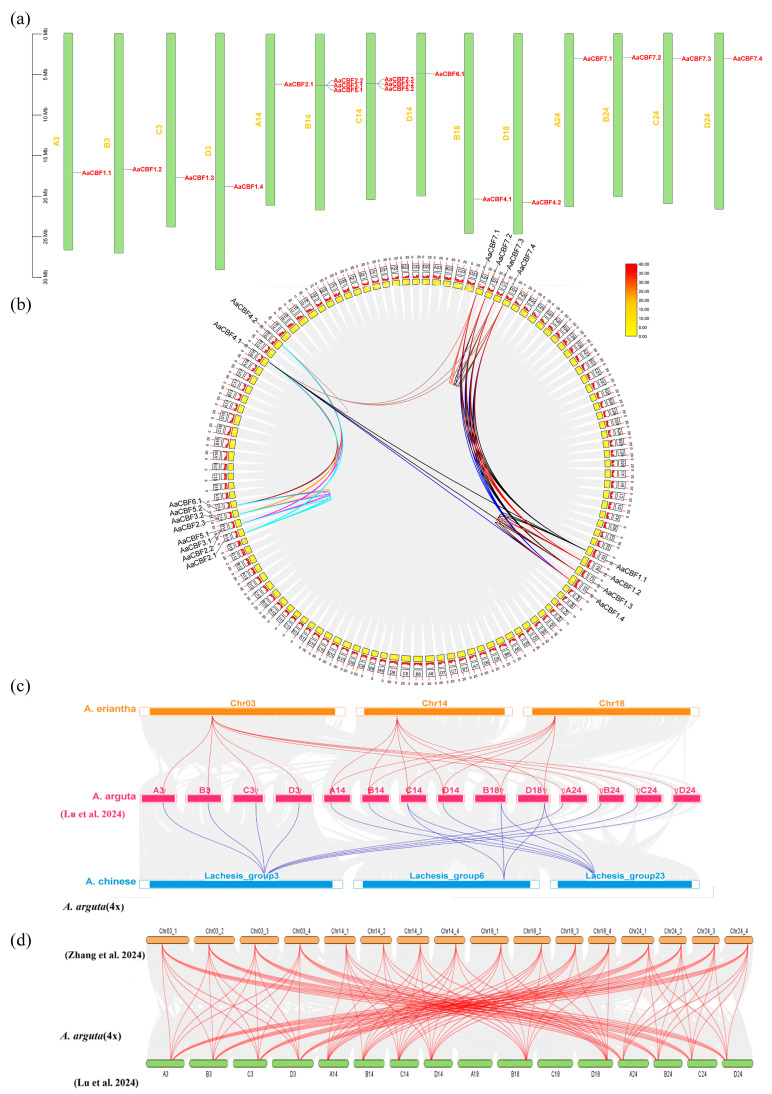
Analysis of chromosomal location and synteny: (**a**) chromosomal location of *AaCBFs* in *A. arguta*; (**b**) synteny of *AaCBFs* in *A. arguta*; (**c**) synteny of *AaCBFs* in *A. chinensis*, *A. eriantha*, and *A. arguta* [29]; (**d**) synteny of *AaCBFs* from different genomes of *A. arguta* [29,33].

The chromosomal locations of the *AaCBF* genes were analyzed. The 18 *AaCBF* genes were unevenly distributed on the chromosomes of Chr3, Chr14, Chr18, and Chr24. ChrB14 and ChrC14 contained the greatest number of *AaCBF* genes, each of which contained three *AaCBF* family members. The remaining *AaCBF* genes were evenly distributed among the other chromosomes (Figure 5a). These differences in gene distribution determine the complexity and diversification of *AaCBFs*, contributing to their evolution.

The collinearity analysis of the *AaCBF* gene family within *A. arguta* revealed that 49 pairs of *AaCBF* genes were collinear. Moreover, the analysis revealed that *AaCBF4.1* was the gene with the highest frequency of collinear events, with collinear relationships identified among 11 *AaCBF* genes. *AaCBF1.1*, *AaCBF1.3*, *AaCBF1.4*, *AaCBF7.1*, *AaCBF7.3*, and *AaCBF7.4* exhibited collinear relationships with 8 *AaCBF* genes. In addition, *AaCBF1.2* and *AaCBF7.2* exhibited collinear relationships with 7 *AaCBF* genes, whereas *AaCBF2.1*, *AaCBF2.2*, *AaCBF2.3*, *AaCBF6.1*, and *AaCBF4.2* exhibited the fewest collinear relationships, with only 5 genes (Figure 5b). These results revealed that these *AaCBF* genes have played important roles in the expansion of the *CBF* gene family in *A. arguta*.

The collinearity analysis revealed the *CBF* genes of *A. chinensis* [31], *A. eriantha* [32], and *A. arguta* [29] had collinear relationships (Figure 5c), indicating that the evolution of homologous *CBF* genes in *A. arguta* was similar to that in *A.eriantha* and *A. chinensis*. In addition, these identified *AaCBF* genes had a high collinearity with the *CBF* genes in the published *A. arguta* genome [33] (Figure 5d), which further demonstrated the accuracy of the identified *AaCBF* genes.

### 3.6. Expression Analysis of AaCBF Genes Under Low-Temperature Treatment and Tissue-Specific Expression Analysis of AaCBF4

**Figure 6:**
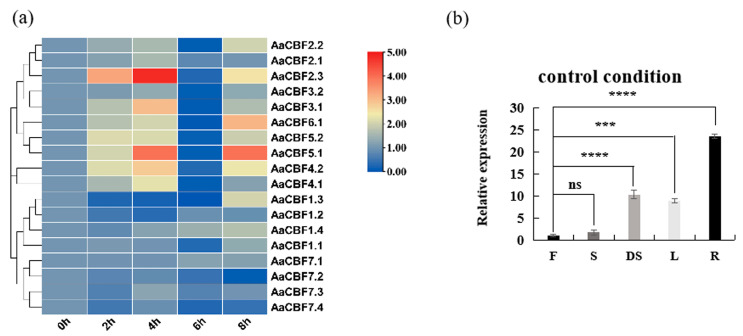
Expression analysis of *AaCBFs* in *A. arguta*. (**a**) Expression of the *AaCBF* genes in dormant shoots of *A. arguta* under cold stress, and (**b**) The expression of Aa*CBF4* in the flowers (F), shoots (S), dormant shoots (DS), leaves (L), and roots (R) of the *A. arguta.* Compared with the flowers group, the asterisks above the column indicate significant differences and extremely significant differences(ns: not significant; *** *p* < 0.001; **** *p* < 0.0001).

*CBF* genes are crucial for the response of plants to cope with chilling stress. Therefore, we examined the expression of *CBF* genes in *A. arguta* under low-temperature conditions (Table S2). RT-qPCR analysis revealed that the expression of CBF genes were induced by low temperature (Figure 6a). The expressions of *AaCBF3.1*, *AaCBF5.1*, *AaCBF2.3*, *AaCBF4.1*, and *AaCBF4.2* were upregulated after exposure to low temperature for 4 h. The expression of the *AaCBF* genes located on Chr3 and Chr24 did not change significantly under cold conditions. Most *CBF* genes presented the lowest expression at 6 h. In addition, the expression of most of the CBF gene family members increased after 8 h of low-temperature treatment. These results indicated that the *CBF* genes in *A. arguta* had different expression patterns during different time points under low-temperature conditions and that the CBF proteins play indispensable roles in enhancing the cold acclimation process of *A. arguta*. In addition, on the basis of our previous study, we selected the *CBF* gene *AaCBF4*, which can regulate the expression of *AaBAM3.1*, and compared the amino acid sequence of the *AaCBF4* gene with that of *CBF* gene family members. The results revealed that the *AaCBF4* gene in the *CBF* gene family was *AaCBF4.1* (Figure S1). The expression pattern analysis revealed that the *AaCBF4* gene was expressed in the flowers, branches, dormant branches, leaves, and roots of *A. arguta*. In contrast to the expression of the *AaCBF4* gene in flower tissue, there was no significant difference in the expression of the target gene in branches, but it was significantly expressed in branches, leaves, and roots during dormancy. The expression level in the dormant branches of *A. arguta* was greater than that in the branches, while the expression level of the *AaCBF4* gene in the roots was the highest, which was 23.5 times greater than that in the flowers (Figure 6b).

### 3.7. Overexpression of AaCBF4 Enhances the Cold Tolerance of A. thaliana

**Figure 7:**
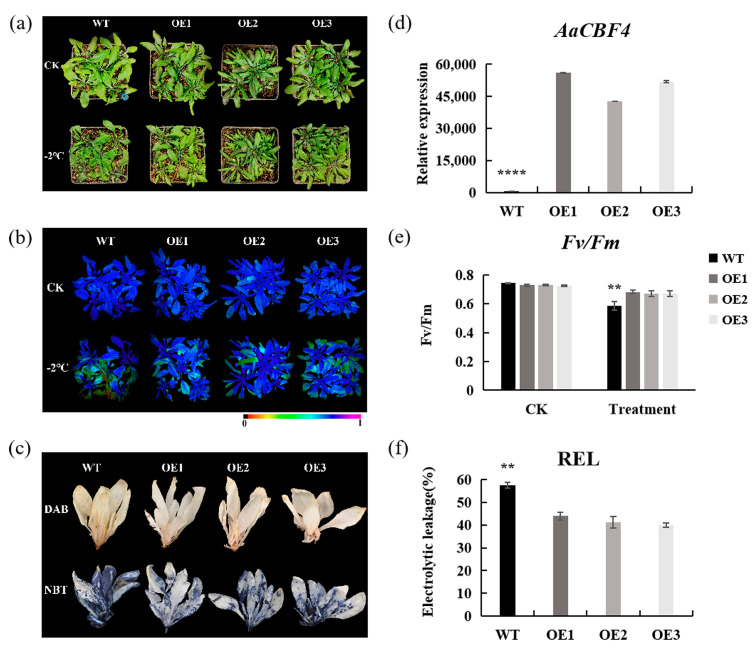
Overexpression of *AaCBF4* enhances the cold tolerance of *A. thaliana:* (**a**) phenotypes of OE1, OE2, OE3, and WT after no treatment (CK) and treatment at −2 °C for 4 h, (**b**,**e**) *Fv/Fm* of the transgenic plants and WT plants after no treatment (CK) and treatment at −2 °C for 4 h (Treatment), (**c**) diaminobenzidine (DAB) and nitro blue tetrazolium (NBT) staining, (**d**) quantitative expression level of *AaCBF4* in *A. thaliana* under cold stress, and (**f**) relative electrolyte leakage (REL). Statistically signifcant diferences compared with WT lines under cold conditions (** *p* < 0.01, **** *p* < 0.0001).

Since *AaCBF4* responds to cold stress, we subsequently used transgenic technology to explore the function of the *AaCBF4* gene. We overexpressed *AaCBF4* in *A. thaliana* under the control of the 35S CaMV promoter. We selected three overexpression lines (OE1, OE2, and OE3) to evaluate the cold tolerance of *A. thaliana* (Figure S2). Phenotypically, there was no significant difference between the *AaCBF4* overexpression lines and the wild type (WT) under normal conditions, but when the plants were treated at −2 °C for 4 h, the leaves of the WT plants exhibited severe wilting and curling, which was more obvious than that in the OE plants (Figure 7a). Moreover, the expression of *AaCBF4* in the three transgenic OE lines was significantly greater than that in the WT line under low-temperature treatment (Figure 7d). To reflect the leaf status and induced cell damage after cold stress, the chlorophyll content, fluorescence parameters, and relative electrolyte leakage (REL) of WT and OE plants after cold treatment were measured. The Fv/Fm ratios of the OE lines were significantly greater than that of the WT, and the RELs of the OE lines was significantly lower than that of the WT, indicating that the degree of cell damage in the OE lines was lower (Figure 7b,e,f). The concentration of reactive oxygen species (ROS) in plants increases after low-temperature treatment, and excessive ROS levels cause cellular damage. Compared with those of OE plants, the diaminobenzidine (DAB) and nitro blue tetrazolium (NBT) staining areas of WT leaves were more evident, and the staining intensity was stronger (Figure 7c). These findings indicated that H_2_O_2_ and O_2_^−^ levels were significantly lower in the OE plants than those in the WT plants after low-temperature treatment. These results indicated that the overexpression of *AaCBF4* in *Arabidopsis* may weaken the accumulation of ROS in vivo and inhibit the damage to the cell membrane after cold stress.

**Figure 8:**
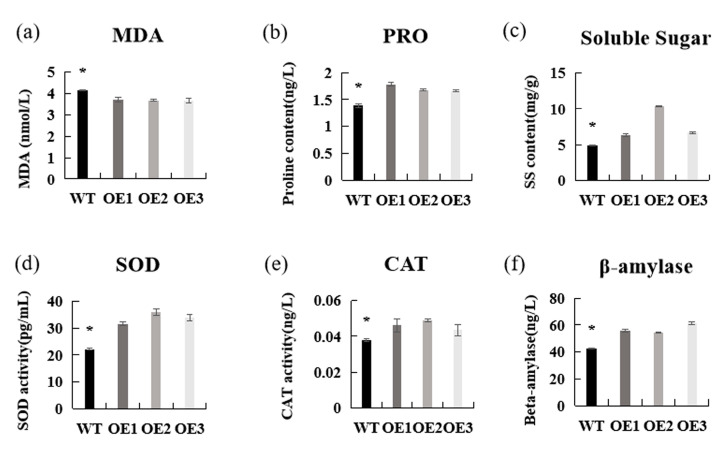
Determination of physiological and biochemical indices in the *AaCBF4*-overexpressing plants and WT plants after low-temperature treatment: (**a**) MDA content, (**b**) proline content, (**c**) soluble sugar content, (**d**) SOD activity, (**e**) CAT activity, and (**f**) β-amylase activity. Comparison of the average data of the transgenic plants and WT plants under low-temperature stress among the three replicates; the error bars represent SEs, and the differences are represented by the asterisk.

To further verify the cold resistance of the transgenic plants, the malondialdehyde (MDA), proline, and soluble sugar levels of *AaCBF4*-overexpressing plants and WT plants after low-temperature treatment were determined. The results revealed that the proline and soluble sugar contents of the OE lines were evidently greater than those of the WT lines, whereas the MDA content exhibited was the opposite trend (Figure 8a–c). These results indicated that the structure and function of the cell membranes in the WT plants were more severely disrupted than those in the *AaCBF4*-overexpressing transgenic Arabidopsis plants. Moreover, after low-temperature stress, the catalase (CAT) and superoxide dismutase (SOD) activities of the OE lines were significantly greater than those of the WT lines (Figure 8d,e). The results revealed that overexpression of the *AaCBF4* gene in *A. thaliana* could eliminate intracellular ROS by increasing the SOD and CAT enzyme activities. The activity of β-amylase in the plant was also determined. The measurement results revealed that the elevated expression of the *AaCBF4* gene in Arabidopsis increased the β-amylase activity (Figure 8f). In summary, overexpression of *AaCBF4* reduced the levels of ROS, REL, and MDA; increased the β-amylase activity and antioxidant enzyme activity; increased proline and soluble sugar contents; and improved the cold resistance of the plants.

## 4. Discussion

Low temperature is an environmental factor that has a significant effect on the growth, productivity, and survival of plants and limits the geographical distribution of various plant species [13]. To cope with low-temperature stress, plants have evolved the ability to adapt to low temperatures, which is known as “cold acclimatization” [34]. CBF transcription factors play important roles in cold adaptation mechanisms. In *Medicago truncatula*, *MtCIR2* regulates cold tolerance by altering the expression and sugar metabolism of *MtCBF/DREB1* [35]; in *Cucumis melo*, *CmABF1* and *CmCBF4* interact with the promoter of *ADC* to improve cold resistance [36]; and in *C. sinensis*, *CsCBF1* interacts with the promoter of the downstream gene *CsLUX* and participates in the cold stress response [37].

*CBF* genes have been identified and analyzed in numerous species. For example, in *Taraxacum kok-saghyz*, ten *CBF* genes were identified as the members of the *CBF* family [8]; sixteen *CBF* genes were found in three species of *Acer*, of which five were *ApseCBFs*, four were *AcyanCBFs*, and seven were *AtuCBFs* [38]; and eighteen *VviCBF* genes were identified in the grapevine genome [23]. However, members of the *CBF* family have not been previously identified in kiwifruit. In this study, eighteen *CBF* genes were identified in the genome of *A. arguta*. The prediction of the subcellular localization revealed that the *AaCBF* genes products could play a role in the nucleus and cytoplasm (Table 1), and the theoretical isoelectric points of the AaCBF proteins were less than seven, which are the same as the isoelectric points reported for the proteins from *Juglans regia* L. [39] and *T. kok-saghyz* [8], indicating that the proteins encoded by the *AaCBF* genes play roles in acidic environments. The results of the phylogenetic tree analysis revealed that, in terms of evolutionary relationships, the *AaCBFs* in Group III were closely related to *CsCBF4*, and the *AaCBFs* in Group V were closely related to *CsCBF1*, *CsCBF2*, *CsCBF3*, *CsCBF5*, and *VvCBF4* (Figure 2). According to previous reports, *CsCBF1*, *CsCBF2*, *CsCBF3*, *CsCBF4*, and *CsCBF5* can respond to low temperatures [37], and the transcription of *VvCBF4* can be induced at low temperatures [40], suggesting that the *AaCBFs* of Group III and Group V may also participate in the response to low temperatures.

Differences in the conserved motifs, conserved domains, and gene structures of *CBFs* may lead to differences in their functions and physicochemical properties [41]. Genes clustered in the same branch of the phylogenetic tree presented similar gene structures (exon/intron numbers), conserved domains, and conserved motifs (Figure 3). However, there was also a certain diversity among *CBF* genes in this species. For example, there were differences in the gene structures and conserved motifs of AaCBF3.1 and AaCBF3.2, AaCBF5.1 and AaCBF5.2 (Figure 3). These results suggested that the *CBF* genes were conserved and diversified during evolution.

The promoter is located the upstream in genes and is an important component of the regulation of gene expression. Analysis of the cis-acting elements in the promoter of the *AaCBF* genes in *A. arguta* showed that there were many light-responsive elements in the promoter, indicating that light may influence the expression of the *AaCBF* genes. Light signals can induce the expression of *CBF* genes, which activate downstream *COR* genes to participate in the cold stress response [42,43,44,45]. In addition, the promoter of the *AaCBF* genes in *A. arguta* also contains a large number of hormone response elements, including ABA response elements, MeJA response elements, and so on (Figure 4). ABA is a plant hormone; endogenous ABA accumulates under cold stress [46,47], and exogenous ABA can enhance the cold resistance of plants [48,49]. Jasmonic acid (JA) and its derivative methyl jasmonate (MeJA) can participate in the ICE-CBF transcriptional regulatory signaling cascade to increase plant cold tolerance [50], and exogenous application of JA also enhances plant cold tolerance [51]. Those indicated that *CBF* genes may be involved in regulating plant adaptation to low-temperature environments. The promoter of some *AaCBF* genes contain anaerobic and low-temperature response cis-acting elements, suggesting that these genes might play a role under stress conditions such as flooding and low temperatures. The discovery of these cis-acting elements further illustrated that these *AaCBF* genes may play a significant role in the cold stress response of *A. arguta*.

The *AaCBF* genes are located on chromosomes Chr3, Chr14, Chr18, and Chr24, and there are three tandem repeat genes distributed on Chr14B and Chr 14C (Figure 5a). Tandem repeats of *CBF* genes also occur in other species, such as *Liriodendron chinense* [41], *Populus alba* [52], and *A. thaliana* [53]. The difference in gene distribution determined the complexity and diversification of *CBFs* in *A. arguta*, which can be attributed to differences in the structure and size of the chromosomes. The linear analysis revealed 49 repeated collinearity events in *A. arguta*, which occurred in 18 members of the *AaCBF* gene family (Figure 5b), further confirming the existence of tandem repeats on the chromosome. In addition, the collinearity analysis of *A. eriantha*, *A. chinensis*, and *A. arguta* revealed the close homologous evolutionary of the *CBF* genes. In previous reports, Zhang et al. [33] identified 34 *CBF* genes in *A. arguta*, whereas our study revealed only 18 *AaCBF* family genes. We speculate that this was due to the different geographical locations of *A. arguta*. The *A. arguta* genome used by Zhang et al. was from Anhui, whereas the *A. arguta* genome used in our study was from Hubei. Plants have developed different genomes in the process of adapting to and evolving in different environments. Moreover, the collinearity analysis of the *CBF* genes between different genomes of *A. arguta* revealed that the *AaCBF* genes were primarily located on Chr3, Chr14, Chr18, and Chr24, and there were a large number of tandem repeats appearing on Chr14, indicating their significant role in the expansion of the *CBF* gene family in *A. arguta*.

*CBF* transcription factors can increase plant resistance to low temperatures by activating the *COR* gene under low-temperature conditions [17]. In previous studies, the expression of *CBF* genes in *L. chinense*, *V. vinifera* [54], *C. sinensis* [37,55], and *Solanum lycopersicum* [56,57] was rapidly induced under cold stress. In ‘Hongyang’ kiwifruit [58], a *CBF*-like gene, *AcCBF1*, which can promote expression in kiwifruit after low-temperature pre-storage to reduce the occurrence of cold damage, has been described. In *A. arguta* [28,59], by analyzing the transcriptomic profiles of different genotypes of kiwifruit under low-temperature conditions, the transcription factor *CBF3* was identified as a gene involved in the low-temperature response; moreover, the *AaCBF4*-*AaBAM3.1* module was activated after low-temperature stress to enhance cold resistance. In our work, the RT‒qPCR analysis revealed that seven *AaCBF* genes were significantly upregulated under low-temperature stress (Figure 6a), indicating that these *AaCBF* genes may play important roles in the response to cold stress.

To further identify the role of the *AaCBF4* gene in low-temperature stress in plant, we obtained *AaCBF4*-overexpressing lines of *A. thaliana*. Studies have shown that the relative conductivity under low-temperature treatment can be used to characterize the degree of electrolyte leakage in plant cells, and therefore, it is often used as an important indicator to measure plant cold tolerance [59]. This study revealed that under low-temperature stress, the REL of WT and transgenic Arabidopsis plants increased, and the REL of transgenic *A. thaliana* plants was lower than that of WT plants (Figure 7f), indicating that the *AaCBF4*-overexpressing lines experienced less stress and exhibited greater cold resistance.

On the one hand, plants undergo oxidative stress when subjected to low-temperature treatment, and large amounts of ROS and free radicals are produced in cells. The common ROS in plant cells are H_2_O_2_ and O_2_^−^, and plants rely on the active oxygen-scavenging enzyme system, which includes mainly peroxidase (POD), superoxide dismutase (SOD), catalase (CAT), and glutathione reductase (GR), to scavenge ROS [60,61]. The production of ROS at levels exceeding the scavenging ability of plants leads to the accumulation of ROS in plants. High levels of ROS may accelerate membrane lipid peroxidation and polymerization between membrane proteins, thereby destroying membrane structure and function. In addition, the accumulation of ROS also increases the MDA content in plants, destroying the intracellular enzyme system and metabolic process [62,63]. In this study, DAB and NBT staining experiments that (Figure 7c) revealed that the content of active oxygen scavengers in the *AaCBF4*-overexpressing plants was greater than that in the WT plants, and the ability of the *AaCBF4*-overexpressing transgenic *A. thaliana* plants to scavenge ROS was greater under low-temperature stress. In addition, the MDA content of the plants (Figure 8a) further confirmed that the transgenic lines overexpressing *AaCBF4* were subjected to mild oxidative stress.

On the other hand, osmoregulation is induced under cold stress in plants, which results in the accumulation of intracellular osmotic substances, reduces osmotic potential, prevents cell water loss, helps protect intracellular macromolecular structures, leads to the regulation and stabilization of biochemical reactions, and promotes cell membrane protection [28,64]. The main substances involved in osmotic regulation in plants include betaine, proline, and soluble sugars. Proline in plants can induce the expression of osmotic stress-related genes, stabilize proteins and cell membranes, and scavenge ROS to prevent cellular oxidative damage. Zhu [65] reported that an increase in the endogenous proline content was positively correlated with improved cold tolerance. Carbohydrates (sucrose, glucose, and fructose) also play key roles in protecting plant cells from low-temperature stress. A previous study revealed that soluble sugars, as osmotic affinity regulators, can accumulate at significant levels in rice seedlings under low-temperature treatment and then participate in maintaining water levels in plant cells and enhancing the cold resistance of rice [66]. β-Amylase is a major hydrolase that can increase the frost resistance of plants by degrading the nonreducing end of starch and releasing soluble sugars [67]. In this study, the greater β-amylase activity observed in the *AaCBF4*-overexpressing transgenic lines than in the WT lines demonstrated that this gene promoted the accumulation of soluble sugars. Moreover, the high contents of proline and soluble sugars observed at low temperatures indicated that the osmoregulatory ability of the *AaCBF4*-overexpressing lines was stronger than that of the WT plants. In summary, the results of this study revealed that the expression of the *AaCBF4* gene was induced by low-temperature stress and that the overexpression of the *AaCBF4* gene in transgenic *A. thaliana* plants increased the tolerance to low-temperature stress. Currently, cold stress remains a major factor in limiting the development of the industry of kiwifruit. Therefore, cultivating low-temperature-resistant kiwifruits are an important and long-term goal. Compared to traditional breeding, genetic engineering can significantly improve breeding efficiency. In this study, the *AaCBF4* gene has been identified as a key gene for cold resistance. In the future, the *AaCBF4* gene can be introduced into the kiwifruit to achieve stable overexpression of the *AaCBF4* gene in transgenic kiwifruit, thereby enhancing its cold resistance and obtaining new germplasm with higher cold resistance.

## 5. Conclusions

In summary, 18 *CBF* gene family members were identified in the genome of *A. arguta*, and structural analysis revealed that the *CBF* gene family members contained two highly conserved motifs (motifs 1 and 2) and an AP2 domain. The expression profile of *CBF* genes in *A. arguta* revealed that the expression of several *CBF* gene family members could be induced at different time points under low-temperature conditions. Taken together, the results of this study and previous studies revealed that the *AaCBF4* gene (*AaCBF4.1*) could respond to low-temperature stress. In addition, the results revealed that the overexpression of *AaCBF4* in *A. thaliana* enhances the cold tolerance of the plants by regulating the activity of antioxidant enzymes and the scavenging of metabolites (proline and soluble sugars) by ROS. Therefore, these results provide a preliminary understanding of the cold adaptation mechanism mediated by the *AaCBF4* gene in kiwifruit in the future and provide a theoretical basis for the cultivation of low-temperature-resistant kiwifruit varieties.

## Figures and Tables

**Table 1:** Identification of *CBF* genes in *A. arguta*.

Gene Rename	Gene ID	AA ^1^	MW ^2^ (kDa)	PI ^3^	SL ^4^	CDS (bp)
AaCBF1.1	Aa3Ag127775.1	233	25,329.31	5.73	Cytoplasm, Nucleus	702
AaCBF1.2	Aa3Bg129754.1	233	25,256.25	5.57	Nucleus	702
AaCBF1.3	Aa3Cg131876.1	233	25,256.25	5.57	Nucleus	702
AaCBF1.4	Aa3Dg133631.1	233	25,355.39	5.73	Nucleus	702
AaCBF2.1	Aa14Ag22350.1	246	27,075.21	5.08	Cytoplasm, Nucleus	741
AaCBF2.2	Aa14Bg23621.1	246	27,211.35	5.02	Cytoplasm, Nucleus	741
AaCBF2.3	Aa14Cg24978.1	246	27,216.33	4.95	Cytoplasm, Nucleus	741
AaCBF3.1	Aa14Bg23622.1	241	26,648.55	4.78	Cytoplasm, Nucleus	726
AaCBF3.2	Aa14Cg24979.1	202	22,291.74	5.14	Nucleus	609
AaCBF4.1	Aa18Bg48528.1	238	26,195.3	5.25	Cytoplasm, Nucleus	717
AaCBF4.2	Aa18Dg51546.1	233	25,569.66	5.57	Cytoplasm, Nucleus	702
AaCBF5.1	Aa14Bg23624.1	270	30,354.12	5.84	Cytoplasm, Nucleus	813
AaCBF5.2	Aa14Cg24980.1	235	26,016.1	5.74	Cytoplasm, Nucleus	708
AaCBF6.1	Aa14Dg26136.1	235	26,178.26	5.14	Cytoplasm, Nucleus	708
AaCBF7.1	Aa24Ag89204.1	232	25,103.11	6	Nucleus	699
AaCBF7.2	Aa24Bg90586.1	232	25,103.11	6	Nucleus	699
AaCBF7.3	Aa24Cg91985.1	232	25,072.06	6	Nucleus	699
AaCBF7.4	Aa24Dg93424.1	232	25,072.06	6	Nucleus	699

^1^ Number of amino acids, ^2^ Molecular weight, ^3^ Isoelectric point, ^4^ Subcellular localization.

## Data Availability

The dataset used in this study is accessible within the article itself and its Supplementary Materials.

## Supplementary Materials

The following supporting information can be downloaded at: https://www.mdpi.com/article/10.3390/life15020227/s1, Figure S1. The amino acid sequence alignment results of *AaCBF4* gene and *CBF* gene family members identified in *A. arguta* genome; Figure S2. Positive trangenic screening of overexpressed *AaCBF4* lines in *A. thaliana*; Table S1. Primers used in this study; and Table S2. The expression of AaCBFs under cold stress.
