# Supplementary material for: Genome-Wide Identification and Expression Divergence of CBF Family in Actinidia arguta and Functional Analysis of AaCBF4 Under Cold Stress
Source: Life (Basel). 2025 Feb 4;15(2):227. doi: 10.3390/life15020227 (PMC11856347; doi:10.3390/life15020227)
Supplement: Supplementary file 1 [file life-15-00227-s001.zip › life-3382250-supplementary.pdf]

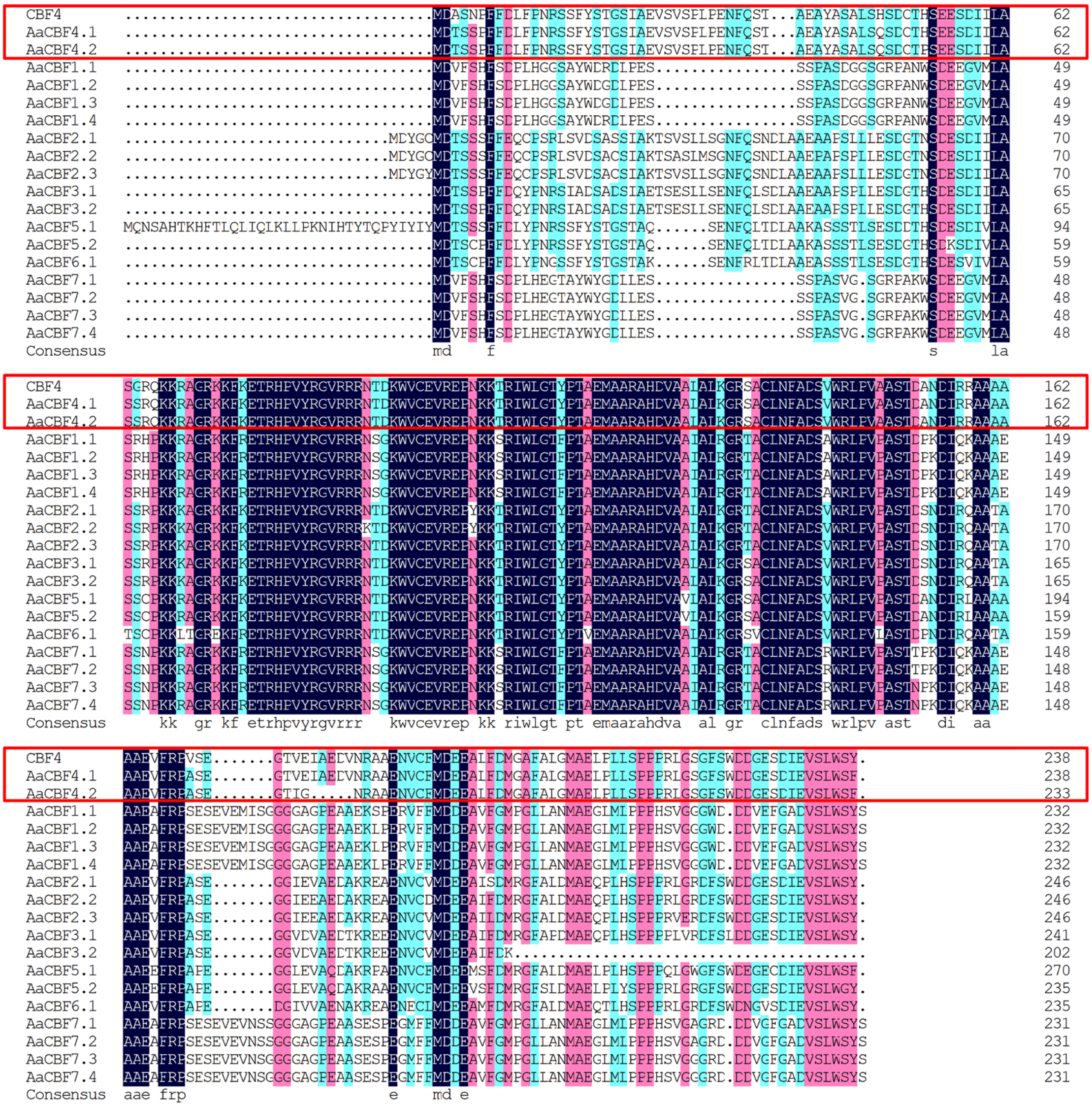

Figure S1. The amino acid sequence alignment results of *AaCBF4* gene and *CBF* gene family members identified in *A. arguta* genome.

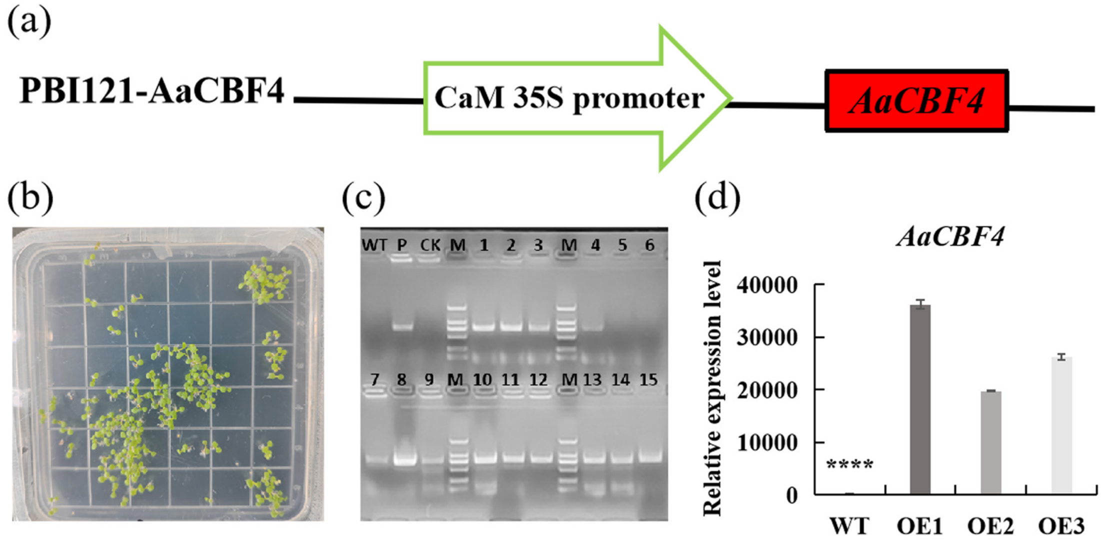

Figure S2. Positive trangenic screening of overexpressed *AaCBF4* lines in *A. thaliana*. (a) Schematic diagram for the construction of the OE-*AaCBF4* vector. (b) Transgenic *AaCBF4* lines seeds in *A. thaliana* were screening with Kanamycin (Kan) (100 mg L-1; Sigma, Japan). (c) Transgenic *AaCBF4* lines were screening by DNA level, M, marker DL2000; 1-15 transgenic lines; WT, wild type plants (negative control); CK, H_2O_2 (negative control); P, recombinant plasmid (positive control). (d) The expression of *AaCBF4* in WT and overexpression lines.
